# Flow and Particle Modelling of Dry Powder Inhalers: Methodologies, Recent Development and Emerging Applications

**DOI:** 10.3390/pharmaceutics13020189

**Published:** 2021-02-01

**Authors:** Zhanying Zheng, Sharon Shui Yee Leung, Raghvendra Gupta

**Affiliations:** 1 Center for Turbulence Control, Harbin Institute of Technology, Shenzhen 518055, China; 2 School of Pharmacy, Faculty of Medicine, The Chinese University of Hong Kong, Shatin, New Territories, Hong Kong; sharon.leung@cuhk.edu.hk; 3 Department of Chemical Engineering, Indian Institute of Technology Guwahati, Assam 781039, India; guptar@iitg.ac.in

**Keywords:** dry powder inhalers (DPI), computational modelling, computational fluid dynamics (CFD), discrete element methods (DEM), pulmonary drug delivery, inhalation therapy

## Abstract

Dry powder inhaler (DPI) is a device used to deliver a drug in dry powder form to the lungs. A wide range of DPI products is currently available, with the choice of DPI device largely depending on the dose, dosing frequency and powder properties of formulations. Computational fluid dynamics (CFD), together with various particle motion modelling tools, such as discrete particle methods (DPM) and discrete element methods (DEM), have been increasingly used to optimise DPI design by revealing the details of flow patterns, particle trajectories, de-agglomerations and depositions within the device and the delivery paths. This review article focuses on the development of the modelling methodologies of flow and particle behaviours in DPI devices and their applications to device design in several emerging fields. Various modelling methods, including the most recent multi-scale approaches, are covered and the latest simulation studies of different devices are summarised and critically assessed. The potential and effectiveness of the modelling tools in optimising designs of emerging DPI devices are specifically discussed, such as those with the features of high-dose, pediatric patient compatibility and independency of patients’ inhalation manoeuvres. Lastly, we summarise the challenges that remain to be addressed in DPI-related fluid and particle modelling and provide our thoughts on future research direction in this field.

## 1. Introduction

Dry powder inhaler (DPI) is a device used to deliver a drug in dry powder form to the lungs. It has emerged as the preferred device for pulmonary drug delivery since its introduction in the 1970s [1]. DPIs are easier to use and free of propellant when compared with pressurised metered-dose inhalers and the administration time is much shorter than that of nebuliser products. Currently, a wide range of DPI devices are available and in development for clinical use. They can be classified into four groups: single-dose devices in which an individual dose is stored in a capsule and loaded into the inhaler by patients immediately before use (Aerolizer^®^, Handihaler^®^, Breezhaler^®^); single-unit disposable devices with the drug stored in a drug pocket within the device (TwinCaps^®^, Twincer™, resQhaler™); multi-unit pre-metered dose devices that contain a series of blisters or capsules stored within the device (Diskus^®^, Ellipta^®^, Diskhaler^®^); multi-dose reservoir devices in which powder is metered from a storage unit by patients before inhalation (Turbuhaler^®^, Twisthaler^®^, Pressair™) [2]. The dry powder formulations are either carrier-based with micronized drug particles (1–5 µm) attached to coarse lactose carriers (50–200 µm) or drug-based (carrier-free) with the agglomeration of fine drug particles. The choice of DPI device is largely dependent on the dose, dosing frequency, and properties of powder formulations [3].

**Figure 1:**
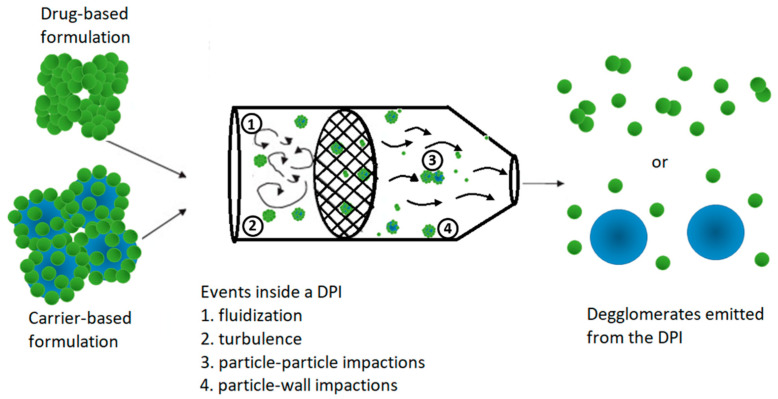
Schematics of dry powder inhaler (DPI) dispersion mechanisms.

Despite the inhaler and formulation designs, patients are required to generate a forceful and deep inhalation through the DPI to de-agglomerate the powder formulation into respirable particles (with an aerodynamic size ≤ 5 µm) for efficient delivery to the lungs. Upon patients’ inhalation through the DPI, the following incidents may affect the particle dispersion: (1) the agglomerate breaks up or detaches from a larger agglomerate due to flow-related forces; (2) the particle/agglomerate collides with other particles/agglomerates, causing it to break up or a drastic change of movement; (3) the particle/agglomerate impacts on the device wall, breaks up into smaller pieces that are entrained back to the air flow or captured by the wall; and (4) the particle/agglomerate reattaches to another particle/agglomerate to form a new agglomerate. The first three events lead to particle de-agglomeration, whereas the fourth event is known as particle re-agglomeration, which has not been frequently discussed in DPI design. Overall, the powder dispersion process in a DPI is highly complex and involve several intertwined physical mechanisms such as air turbulence and shear flow-induced aerodynamic forces, particle-device impactions within the inhaler body, mechanical vibration and particle-particle collisions [4] (Figure 1). The relative importance of these mechanisms varies with the designs of the inhaler and powder properties.

With the rapid advancement in computational technologies, computational fluid dynamics (CFD), discrete particle methods (DPM) and discrete element methods (DEM) have become useful tools to predict the air flow patterns, turbulence levels, particle trajectories and particle deagglomeration within a DPI and the delivery paths of aerosols to the lungs [5]. The total number of particles within the device is very large and a simulation with the consideration of all particles and complex interactions would quickly overwhelm the computational power. This means a major challenge for DPI modelling is to obtain a reasonably accurate prediction of the particle movements with the consideration of only limited representative particles and essential forces. Therefore, validating the simulation results with experimental data would become critical to confirm the relative contributions of the dispersion mechanisms for subsequent DPI and formulation design optimisation.

Conventionally, DPI is limited for the treatment of airway diseases such as asthma and chronic obstructive pulmonary disease (COPD) for patients who are ≥6 years old. In the past decade, there has been an increasing trend to explore new applications of DPI to deliver antimicrobials or other high-dose active ingredients and to deliver medications to paediatric patients. Computational modellings for these emerging applications are relatively scarce. In the present review, we first provide an overview of the capabilities of various conventional and state-of-the-art modelling approaches in predicting the flow and particle behaviours in DPIs. The application of computational modelling is then exemplified in guiding the development of emerging DPI designs, as well as the optimisation of existing DPI design for a wider range of patients. Lastly, we summarise the challenges in computation modelling of DPI and provide our thoughts on future research directions in this field.

## 2. Modelling Approaches of Flows and Particles in DPIs

Computational modelling provides a way to predict the temporal, spatial and size distributions of particles as they leave the DPI. Currently, the fluid flow is often described by mass and momentum conservation equations in an Eulerian framework, and the particle motion is described in a Lagrangian fashion following Newton’s second law. In this section, we briefly introduce various modelling techniques used in DPI-related simulations and their capabilities to predict the flow and particle behaviours in DPIs are then elaborated.

### 2.1. Fluid Flow and Turbulent Models

In a DPI, the flow of continuous gas phase is governed by the mass and momentum conservations equations, which can be solved in a coupled or segregated manner to obtained velocity and pressure field and other parameters of interest. Several numerical techniques such as finite volume method, finite difference method and finite element method have been developed to solve this system of non-linear partial differential equations. Several open-source packages, such as OpenFOAM, and commercial software packages, such as ANSYS CFD, COMSOL, CFD-ACE+, STAR-CCM and TransAT are available to perform the computations.

Due to the complex geometry of the DPI flow passage and the relatively high flow rate, the problem is normally considered to be turbulent, which is characterised by the presence of a wide range of time and length scales in the flow field. Capturing all the turbulent time and length scales requires very fine spatial and temporal discretization (simulations fulfilling these requirements are usually called direct numerical simulation (DNS)), making most computation attempts impractical even with the powerful computational resources available today. For DPI applications, the turbulence is normally not resolved directly but relying on the heuristic turbulence models.

In a simplified approach, the instantaneous flow properties such as velocity and pressure are decomposed in averaged and fluctuating components and averaged to obtain mass and momentum conservation equations, generally termed as Reynolds-averaged Navier–Stokes (RANS) equations, for mean properties. Along with the usual terms in Navier–Stokes equations, an additional term known as Reynolds stress appears and is modelled in terms of other variables to obtain a closed system of equations. This is known as the closure problem of turbulence. Several heuristic models have been developed to describe Reynolds stresses [6]. A common approach is based on the turbulent velocity hypothesis, in which Reynolds stress is proportional to the mean rate of strain and the proportionality constant is termed as turbulent viscosity. The problem thus reduces to the modelling of turbulent viscosity in terms of turbulent quantities. Two commonly used RANS models are *k-ε* and *k-ω* models where *k* is the turbulent kinetic energy, *ε* is the rate of dissipation of the turbulent kinetic energy and *ω* (=*ε*/*k*) is the specific rate of dissipation. To enhance the near-wall modelling performance, Menter [7] proposed a *k-ω* SST model to combine *k-ε* and *k-ω* models using a blending function that is set to zero at regions close to the walls and unity at regions away from the walls. Compared to these models (sometimes called two-equation model), higher-order models, such as Reynolds-stress models, are expected to be more accurate, but require more computational resources and more effort to obtain a converged solution [8].

Most existing DPI studies have employed RANS equations to model the turbulent flow [9]. In recent years, large eddy simulation (LES) is being increasingly adopted for various DPI applications [10,11,12]. LES is a more refined and computationally expensive approach, in which larger eddies are captured directly from the simulations and smaller eddies are filtered using low-pass filters with their flow behaviours modelled using sub-grid scale models [6]. Compared to RANS, LES provides more information of turbulence and vortex structure, especially about the small-scale structures present in the flow field, and therefore helps in describing the particle motion induced by the flow better. On the other hand, the computational time of LES is about one order of magnitude higher than two-equation models [13], which is currently the major obstacle for a wider acceptance of this method. Table 1 summarises the available CFD methods for DPI simulations and relevant applications.

### 2.2. Modelling of Particle Behaviours

The modelling of particle motion is strongly coupled with the modelling of the fluid flow. As the fluid flow is solved using an Eulerian framework, particle motion can be described by either an Eulerian or Lagrangian approach, where the latter is more common in DPI applications. They are often termed as Eulerian–Eulerian and Eulerian–Lagrangian approaches, respectively, with the first ‘Eulerian’ indicating the description of the fluid flow.

In the Eulerian–Eulerian approach, both the gas and solid phases are considered as interpenetrating continua, where both phases can coexist simultaneously in a grid or mesh element and the interface between the phase is not resolved. The conservation equations are solved for each phase. The stresses in the solid phase are obtained considering the random motion of solid particle caused by the inelastic particle–particle collision. The mass and momentum exchange between the two phases are modelled as source/sink terms in the conservation equations for each phase.

In the Eulerian–Lagrangian approach, Newton’s law of motion is solved for the solid particles and the position of the solid particles or envelope/ensemble of solid particles is calculated with time. The natural ability of Eulerian–Lagrangian approach to handle particle inertia and wall collisions makes them more attractive for simulation of inhaler aerosols, although their computational costs are generally higher.

### 2.3. Fluid–Particle and Particle–Particle Interaction

Some Lagrangian-type simulations do not consider the interaction of the particle with its neighbouring particles and assume the particle motion is only caused by gravity, flow-related forces and particle–wall impaction. These simulations are sometimes called discrete particle methods (DPM). The flow-related forces may include drag force, pressure gradient force, virtual/added mass force, Basset force and transverse lift forces. These forces are determined either directly from the flow field results or through empirical correlations. The modelling accuracy depends on the forces considered and the correlations used. In a one-way coupling DPM, the influence of the particle to the flow field is not considered, so the flow field and particle motion can be resolved separately; a two-way coupling DPM describes the effect of particles on the flow using an additional body force term in the flow’s momentum equation.

The DPM method has been widely used to predict the particle dispersion in DPI devices, mainly due to its simplicity and capability of describing the particle trajectories, at least indicatively. However, it is unable to model the particle–particle collision or the particle de-agglomeration, which limits its use for more refined simulations.

The Lagrangian simulations that consider the particle–particle interaction are frequently called discrete/distinct element methods (DEM). The concept of DEM was initially proposed by Cundall and Strack [40], where the translational and rotational motion of a particle is governed by Newton’s laws of motion with the following governing equation [41].

(1) $$m_{i}\frac{dv_{i}}{dt}=\underset{j}{\sum }F_{ij}^{c}+\underset{k}{\sum }F_{ik}^{nc}+F_{i}^{f}+F_{i}^{g}$$

(2) $$I_{i}\frac{d\omega_{i}}{dt}=\underset{j}{\sum }M_{ij}$$

where *v_i_* and *ω_i_* are the translational and angular velocities of particle *i*, respectively, $F_{ij}^{c}$ and $M_{ij}$ are the contact force and torque acting on particle *i* by particle *j* or walls, $F_{ik}^{nc}$ is the non-contact force acting on particle *i* by particle *k* or other sources, $F_{i}^{f}$ is the particle–fluid interaction force on particle *i* and $F_{i}^{g}$ is the gravitational force.

Two different approaches, hard particle [42] and soft particle [40], are used to model contact forces between the particles. In the hard particle approach, particles are assumed to be perfectly rigid, non-deformable during collisions and only binary collision, that is the collision between two particles, can occur. Whereas in the soft particle approach, the particles are geometrically rigid but can deform during the contact. The deformation during the contact is modelled as overlaps between particles.

Mathematically, there are many ways to derive the contact forces based on the geometrical and physical characteristics of the particles, such as the shape, material properties, and moving state of particles [41]. It is generally resolved in normal and tangential components. Some normal contact force models [43] are Hertzian spring [44], damped linear spring [40], hysteretic linear spring [45,46], damped Hertzian spring [47], non-linear damped Hertzian spring, hysteretic non-linear spring and continuous potential models. Common tangential force models are described in Mindlin [48] and Mindlin and Deresiewicz [49]. Typical non-contact forces include the van der Waals force, capillary force and electrostatic force, which become significant for small particle size and separation distance, as typically seen in DPI-related problems.

For particle-fluid flows in DPIs, the DEM can be coupled with CFD to resolve the particle–fluid interaction, which is sometimes called CFD–DEM method. The simulation can be achieved by incorporating DEM equations with popular CFD codes, such as ANSYS Fluent. Some specialized DEM software packages are also available, such as the EDEM, which employs a soft sphere approach and LIGGHTS, an open-source DEM code.

## 3. Review of Up-to-Date DPI Modelling Studies

Recent DPI simulation studies can be classified into the following three categories:

- Particle trajectory modelling: the prediction of particle trajectory with the consideration of only fluid related forces and effects (particles may be assumed as point particles without volume or spheres when particle inertia is considered).
- Particle–particle interaction modelling: considering particle–particle forces in the simulation to study the particle agglomeration and de-agglomeration behaviours.
- Multi-scale modelling: macro-scale modelling of the DPI flow and particle trajectory with the consideration of the micro-scale particle–particle interaction.

Studies belonging to the first category generally use the DPM method to track the motion of the particles/agglomerations in a device-scale. The second category relies on DEM to study the micro-scale particle–particle behaviours. The third category incorporates the features of the first two, which represents a new trend in DPI simulations. These studies are critically reviewed in this section with the simulation details are listed in Table 2.

### 3.1. Particle Trajectory Modelling

**Figure 2:**
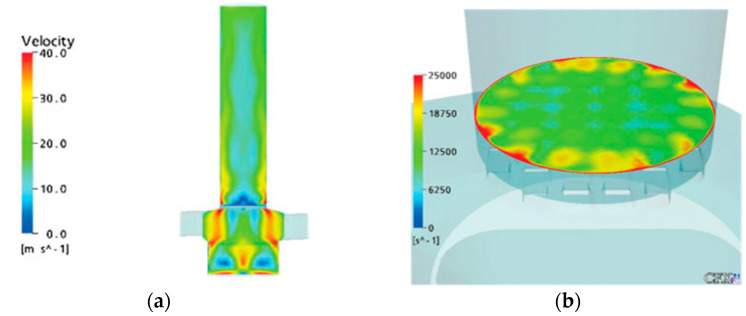
Calculated velocity profile (**a**) and turbulence scale (**b**) of an Aerolizer^®^ using RANS with *k-ε* SST model. Reproduced with permission from [8], Elsevier, 2004.

Coates et al. [8,16,17,18,19] carried out some of the earliest DPI modelling studies, in which particle trajectory tracking was performed as a post-processing operation based on the CFD results of the flow field. A commercial product, Aerolizer^®^, has been used as the inhaler base model and various design modifications have been examined to understand the effect of grid structure, mouthpiece length [8], capsule presence [19], air flow [17], air inlet size [18] and mouthpiece geometry [16]. The flow field in the inhaler was obtained by solving RANS equations and *k-ε* SST turbulent model, as shown in Figure 2. The simulations were carried out using the commercial CFD code ANSYS CFX. The measurement of flow velocity profile at the inhaler outlet was performed by a laser Doppler velocimetry (LDV) to validate the computational results and a reasonably gdoo agreement was obtained between the experiments and simulations, indicating the CFD models were capable of simulating both the axial and tangential flow through the devices. According to the CFD results, reducing the air inlet size or the inhaler grid voidage of the Aerolizer^®^ resulted in an enhanced flow turbulence. An increase of the flow turbulence would increase the chance of particle–particle collision, though it did not mean a necessary increase in the particle–wall collision.

Upon the CFD results, a large number of particles were placed at the upstream of the flow and their trajectories were tracked subject to drag and turbulent dispersion forces caused by the surrounded flow. This Lagrangian particle tracking techniques were performed using a frozen flow field, meaning the flow field did not vary as the particles moved from the initial location to downstream. It was possible that particle–wall impaction would occur, and by setting different walls within the devices to have a zero coefficient of restitution, the frequency and location of impactions could be determined. Each of the tracked particles represented an agglomerate and a more frequent wall impaction therefore resulted in a better de-agglomeration of drug particles. 

**Figure 3:**
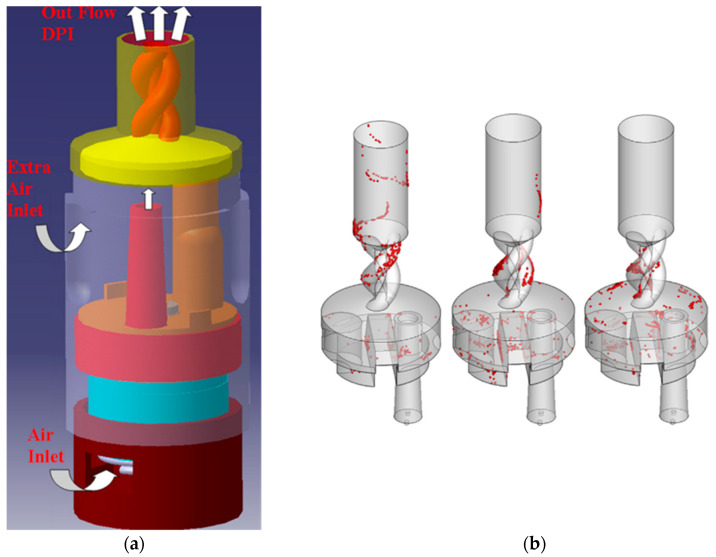
Schematic of a multidose dry powder inhaler (**a**) and calculated particle deposition (particle sizes from left to right are 1 μm, 2 μm and 5 μm, respectively) in the device (**b**) via a simple particle trajectory modelling. Reproduced with permission from [13], Elsevier, 2013.

Milenkovic et al. [13] modelled the steady flow and particle depositions in Turbuhaler^®^ (AstraZeneca), a multidose dry powder inhaler, with the consideration of drag and buoyancy forces imposed onto the particles. The number of particle deposition (a deposition was resulted when the normal collision velocity was less than a critical capture velocity) was obtained based on the particle–wall collision results (Figure 3). Calculations were carried out using the commercial software package ANSYS Fluent for a range of particle sizes (both single-sized and size distributed particles) and the simulated particle deposition was found to be in good agreement with experiments. Milenkovic et al. [21] later extended their study to the dynamic flow and it was found that compared to steady flow simulations, the fine particle fraction (FPF) determined by dynamics flow simulations achieved a better agreement with experimental data in the literature. Modified DPIs based on the commercial device Turbuhaler^®^ were also examined and improved particle deposition and FPF were evident [22].

Wong et al. [23,24,25] utilised a combination of CFD and standardised entrainment tubes to investigate the influence of wall impaction, turbulence and grid structure on the break-up and aerosol performance of a model inhalation formulation, where Lagrangian particle tracking was conducted. The results indicated the importance of impaction, which dominated agglomerate break-up in the dry powder system. 

Similar modelling approaches have been used to examine the performance of other DPIs. Donovan et al. [20] modelled carrier particle trajectories through two commercial DPIs (Aerolizer^®^ and Hanihaler^®^) and it was found that the particle–inhaler collisions, which are an important indicator of the inhaler dispersion performance, were influenced by both the carrier particle size and inhalation device design. Moska and Sosnowski [14] compared the performance of two inhalers, Aerolizer^®^ and its modified product NGC (new generation Cyclohaler^®^). ANSYS Fluent was used to solve the flow field and the turbulence of the flow was modelled using the standard *k-ε* model. It was concluded that the NGC, which had a short mouthpiece section, caused a lower aerosol particle deposition, benefiting the drug delivery to the lower respiratory tract. Shur et al. [28,29] obtained carrier particle trajectories within both unit-dose (Cyclohaler^®^ and Handihaler^®^) and multi-unit dose (Flixotide^®^, Accuhaler^®^ and Multihaler^®^) DPIs via CFD, and found that the variations of the impact velocity and angle could result in different inhalation performance. Suwandecha et al. [26] simulated the flows in a standard Cyclohaler^®^ (or Aerolizer^®^) and three modified devices, which incorporated some features of the Rotahaler^®^. The CFD simulations were performed using ANSYS Fluent and the standard *k-ε* turbulence model was used. The importance of the inhaler grid was emphasised, which straightened the flow streamline and influence the turbulent effect.

Sommerfeld and Schmalfuß [11] later extended the previous Lagrangian particle tracking to account for all possible forces, in which the transverse lift forces were not considered in other studies. The open-source-code OpenFOAM was used to solve the Reynolds-averaged conservation equations, together with the *k-ω* SST turbulence model. A typical swirl-flow inhaler device was considered, which resembled the commercial product Aerolizer^®^. The modelling results showed that when accounting for all fluid forces on the particles, the averaged particle velocities greatly increased and the particle residence time in the DPI was reduced, resulting in a much higher particle–wall impact velocity, and diminished total wall collision number. It was concluded that the transverse lift forces due to shear flow and particle relative rotation needed to be accounted for in Lagrangian calculations of particle motion within inhaler devices.

The possibility of incorporating particle trajectory modelling with physiologically-based pharmacokinetic (PBPK) modelling has also been evaluated by Vulović et al. [61], in which ANSYS Fluent was used to simulate fluid flow in an Aerolizer^®^ and the standard *k-ω* model with low Reynolds number (LRN) was adopted for the turbulent flow. 

Due to the limitations of RANS, simulations might not be capturing all details of the swirling flow generated in the devices, especially the details of the turbulence-related fields. Nevertheless, Milenkovic et al. [13] did find that although significant turbulence non-homogeneity and anisotropy were shown in the flow field, there were only some local differences in the secondary flow prediction between an LES and *k-ω* SST model.

These particle studies have the advantages of relatively low computational cost and the ability to indicatively capture the flow path and wall collisions of carrier particles or agglomerates. On the other hand, due to the absence of particle–particle interaction, such methods are not able to fully describe various important particle behaviours, such as particle–particle collisions and the particle de-agglomeration in DPIs. The level of de-agglomeration can only be speculated indirectly from the wall impaction and flow turbulence.

### 3.2. Particle-Particle Interaction Modelling

The study of particle agglomeration, de-agglomeration or reattachment requires the numerical models to include the effects of particle–particle interaction. In this section, the modelling studies for both particle de-agglomeration and agglomeration are discussed.

#### 3.2.1. De-Agglomeration

In the inhaler, de-agglomeration is one of the most important particle behaviours since it determines the final particle size distribution and hence the FPF. For either carrier-based or carrier-free drug formulation, it is initially presented as the agglomeration form, although the two have different de-agglomeration mechanisms.

Calvert et al. [50,51,52] numerically studied the aerodynamic dispersion of loose aggregate in a uniform fluid flow, and the aggregate could be regarded as a carrier-free powder element in a DPI. The effect of particle cohesion and contact force was described by the JKR model [62], which was strongly correlated with the surface energy and diameter of the aggregate. In their simulation results, there was a relative particle-fluid velocity threshold and once exceeding this threshold, the dispersion occurs quickly and approached a complete dispersed state [51]. The dispersion behaviour of the aggregate depended on the ratio of aggregate diameter to primary particle diameter: if the size ratio was small, the aggregate disintegrated rapidly above the threshold velocity; however, as the size ratio increased, the dispersion behaviour switched to a much slower surface peeling mechanism because more time was required for the force on the surface of the aggregate to propagate inside it [52]. Although these studies were carried out within a simple uniform flow field with only a single aggregate, the dispersion behaviours might resemble those in a DPI with drug-only powder. On the other hand, the study was not specifically for DPI applications but for a general particle de-agglomeration problem where a primary particle size of 100 μm was used, which was significantly larger than actual active pharmaceutical ingredient (API) particles in the inhaler (normally less than 5 μm). Therefore, relevant mechanisms might not be entirely valid for DPI problems.

The de-agglomeration induced by wall impaction can be significantly more intense than that induced by fluid flow. Thornton et al. [54] simulated a dense spherical agglomerate consisting of auto-adhesive particles impacting orthogonally with a target wall and concluded that rebound, fracture or shattering might occur, depending on the magnitude of the impact velocity specified. Most of the input energy was dissipated due to plastic deformation of the microstructure within the impact zone and pre-existing flaws did not have a significant effect on the agglomerate “strength”. Several follow-up studies were also carried out to further study relevant mechanisms and the effect of various parameters on breakage of the agglomerate [55,56,57]. These studies were not specifically targeted for DPI applications but a broader range of process engineering problems, and the primary particle sizes ranging from 16 to 60 μm were used, which were considerably larger than a typical fine drug particle for inhalation. A later study using particles with typical sizes for DPI applications (5 μm) was carried out by Tong et al. [34] and indicated that the damage ratio of agglomerates increased with the increase of impact velocity. The dependence of impact angle was also examined, and it was found an impact angle of 45° resulted in the maximum breakage performance.

In a DPI, the de-agglomeration is induced simultaneously by both aerodynamic forces and impaction. Tong et al. [32] presented an early investigation of powder dispersion based on a combined CFD and DEM approach to model the agglomerate behaviour of different particle sizes in a cyclonic flow, where the flow field resembled a swirling chamber of the commercial DPI Cyclohaler^®^ (Aerolizer^®^). The results indicated that the powder dispersion was mainly governed by the particle–wall impact and the internal shearing induced by air flow only played a minor role within the system. Tong et al. [31] further studied the powder de-agglomeration in different customised impaction throats, and an improved dispersion performance was found for the throats with two angles, where two impactions occurred: the first caused the major damage to the agglomerate while the second generated more fine particles with a size less than 5 μm. A complete Cyclohaler^®^ was later adopted where the powder dispersion mechanisms were investigated through a coupled CFD and DEM approach. It was found that multiple major impactions occurred between the agglomerates and the chamber wall, which fragmented the agglomerates into large pieces without generating many fine particles and the subsequent impactions between the fragments with the grid were identified as the key factor for the FPF [35]. It was possibly the first study to apply the CFD–DEM method in carrier-free DPI applications.

**Figure 4:**
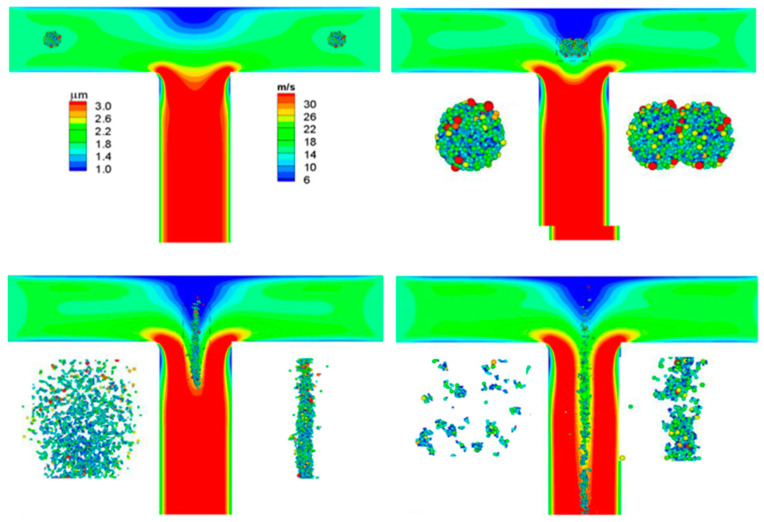
Breakage pattern of agglomerate due to collision simulated by Tong et al. [37] at the progression time. Reproduced with permission from [37], Elsevier, 2016.

A recent development in this field is the detailed modelling of agglomerate–agglomerate collisions, which have not been well described in the past. Tong et al. [37] simulated the collision between two mannitol agglomerates in a T-shape pipe, as shown in Figure 4. The results showed that the collision between agglomerates had a significant effect on the aerosolisation process and there was a threshold agglomerate velocity, above which would increase aerosolisation and otherwise would decrease. Dizaji et al. [63] examined the collision of two agglomerates in a shear flow and indicated that depending on the value of the adhesion parameter and the ratio of offset distance to agglomerate radius of gyration, the collision could result in an agglomerate merger, bouncing and fragmentation. A study by van Wachem et al. [39] proposed a new dimensionless parameter to capture the properties of the agglomerate and the collision, leading to a satisfactory fit of the micro-scale collision modelling results.

The aforementioned studies are related to carrier-free agglomerates, but de-agglomeration modelling specifically for carrier-based agglomerates has also been carried out since the 2010s [33,58,59,64,65]. Yang et al. [58] employed the CFD–DEM approach to study the dispersion of a carrier-based agglomerate in a uniformed air field and it was observed that when the air flow was introduced, the fine API particles in the downstream regions were removed from the carrier directly. The API particles in the middle regions firstly moved to the downstream regions and then detached from the carrier; most API particles in the upstream regions were not removed. The dispersion process during the impact of a carrier-based agglomerate and a wall was later modelled using DEM, and two dispersion stages were found: a fast dispersion stage and a stabilised stage. Particle dispersion induced by particle–wall impaction for carrier-based formulation could be well approximated using the cumulative Weibull distribution function governed by the ratio of the overall impact energy and adhesion energy [59]. Nguyen et al. [64] studied the collision between a carrier coated with fines and a non-coated carrier and investigated the transfer of fine particles between carrier particles. Fine-particle fragments were restructured after a collision and those transferred to another carrier had higher tensile strength than the original fragments. Tong et al. [33] simulated the aerosolisation process of carrier-based formulations with high drug loadings after impaction with a target wall. The results showed that increasing the carrier-drug mass ratio could improve aerosolisation performance. A smaller carrier was preferred to satisfy the requirements of drug loading and FPFs at the same time. Ariane et al. [65] used DEM to simulation the dispersion process induced by a collision between a carrier-based agglomerate with both elastic and sticky walls. The effect of agglomerate rotation was discussed, which was of importance if the agglomerate kinetic energy resulting from the rotation was below that of the translational velocity. An elastic or sticky wall had only a marginal effect on the dispersion ratio. A simplified analytic model was proposed, which could be used as a sub-scale model in full-scale inhaler dispersion modelling. Tamadondar and Rasmuson [66] recently discussed the effect of carrier surface roughness for collision-induced dispersion and found that at the rebound, most remaining fine particles fall into the particle cavities and establish multiple contacts with the carrier surface.

Cui et al. [67,68,69] took a different approach to quantify the detachment rate of the fine drug particles from the carrier by using a fully resolved Lattice–Boltzmann method (LBM). A micro-scale model was initially established based using LBM, which included a spherical carrier particle randomly covered with a monolayer of fine spherical powder. The particle cluster was centrally fixed in a cubic flow domain. The fluid forces in the fine particles as a function of their position and flow condition were then obtained, which could be used to determine the probability of their detachment from the carrier [67]. The LBM was later tested by comparing the simulated drag and life force coefficients with analytical solutions and a good agreement was achieved [68]. Cui and Sommerfeld [69] further extend the LBM to account for the influence of turbulence on the possibility of drug particle detachment.

#### 3.2.2. Agglomeration

Compared to de-agglomeration, the agglomeration behaviour in the inhaler has not been frequently discussed. Although it is mainly the dispersion process of drug powder that determines the final FPF, there is still a chance that the dispersed particles re-agglomerate before exiting the inhaler device. More attention needs to be paid to the study of relevant agglomeration mechanisms.

Several studies have been carried out for the purpose of numerically creating agglomerates which could later be used for particle dispersion simulation. Thornton et al. [70] used a DEM approach to generate a large agglomerate from 1000 monodisperse primary particles with a radius of 100 μm. All primary particles were initially distributed within a prescribed region and then a centripetal gravity field was imposed to bring the particles together. The surface energy was later applied with small increments to the required level up to 3.0 J/m^2^. Then the centripetal gravity field was gradually removed, and a stable agglomerate was formed. Similar methods have been widely used for the preparation of agglomerates for subsequent DEM studies [34,51]. Although the above approach has successfully generated stable agglomerates that resemble those in actual drug powder, it is not the natural way by which the agglomerate is formed in a DPI. In an actual inhalation process, new agglomerates are most likely formed due to the re-attachment of dispersed fine particles.

Afkhami et al. [71], on the other hand, discussed the actual agglomeration process of particles in a turbulent channel flow and the influence of particle surface energy and fluid turbulence on the agglomeration. Their results indicated that the turbulent structure of the flow dominated the motion of the particles, creating particle–particle interactions, and a positive relationship between particle surface energy and agglomeration was observed. Two separate regions with enhanced particle agglomeration were found: in the near-wall region due to the high particle concentration, and in the high turbulence regions close to the walls caused by the shearing effect of the flow at the no-slip boundaries. Afkhami et al. [72] further examined the effect of turbulence levels and particle cohesion levels on the agglomeration behaviour in particle-fluid flow. The results suggested that the rate of agglomeration was strongly influenced by the intensity of the flow turbulence: for high surface energy particles, it increased with an increase in flow turbulence intensity; for low surface energy particles, it diminished with an increase in flow turbulence intensity.

The formation of carrier-based agglomerates has also been simulated as an initial step for fine particle dispersion modelling. In the DEM study carried out by Yang et al. [58,59], a carrier particle, with several drug particles randomly positioned around it, was generated and then the drug particles were set to move towards the centre of the carrier at a specified velocity until they attached to the carrier. The adhesion between the carrier particle and drug particle was initially set to a relatively high value to allow an easier attachment and then reduced to the required value that was used in the simulation. Yang et al. [60] took a different approach by setting electrostatic forces between the carrier and drug particles. Initially, a carrier particle was generated at the centre of a cubic container while several drug particles were randomly generated in the empty space of the container. The carrier particle was positively charged, the drug particles were negatively charged, and they were then mixed by vibrating the container with a periodic velocity. The number of drug particles attached to the carrier particle increased with time until the moving direction of the container changed. During the vibration, the carrier particle impacted the wall of the container, causing drug particles to detach from the carrier, and then attracted to the carrier again under the influence of electrostatic force. Simulation results indicated that the charge of the carrier particle played a significant role in the electrostatic interactions between carrier and API particles. The mean contact number of drug particles with the carrier particle increased with increasing charge and decreased with increasing vibrational velocity amplitude and frequency. It could give a better understanding of the mixing process of carrier and drug particles in DPI application. Although these simulations were only performed for a single carrier particle, they could indicate the mechanism of particle agglomeration in the actual DPI process.

In general, the CFD–DEM method has been increasingly used to simulate the micro-scale de-agglomeration and agglomeration behaviours for both carrier-free and carrier-based formulations, and it has been proven to be a promising approach with great potential in the design of high-performance DPI.

### 3.3. Multi-Scale Modelling

Recently, more comprehensive studies of the DPI particle behaviours with multi-scale modelling have been increasingly reported. This approach not only includes the micro-scale particle–particle interaction but also integrates the micro-scale effect into the device-scale (macro-scale) flow and particle trajectories, so that the FPF of the DPI can be obtained directly from the modelling.

Tong et al. [30] proposed two-stage modelling to predict powder aerosolisation in carrier-based DPIs based on CFD-DEM. In the first stage, a large carrier particle randomly covered by a number of drug particles was generated and put into a wall-bounded shear flow and impacted onto a target wall at a certain velocity and impact angle. Through more than 200 simulations, the relationship between the number of fine particles released from the carrier and wall deposition with impact angle and velocity was determined. Empirical equations were then developed to link the detachment performance. In the second stage, the dynamics of the carrier particles in the commercial inhaler, Aerolizer^®^, was simulated using CFD–DEM. When the carrier particles collided with each other and with the device wall, the impact information of carrier particles was recorded. The drug particles were not included in the simulations and they were assumed to follow the flow streamline and their detachment from the carrier particles could be determined by the empirical equations developed in the first stage. The combination of all drug particle detachment behaviour determined the overall dispersion performance of the inhaler. Leung et al. [36] used the same approach to study the effect of different grid structure on the aerosol performance of DPIs. The results showed that the effect of inhaler design on the aerosolisation of DPIs was highly dependent on the properties of formulations.

Van Wachem et al. [12] proposed an approach to combine microscale, meso-scale and macro-scale modelling to describe the particles and the dynamic nature of the flow in an inhaler. At the micro-scale, the interactions of the small active drug particles with larger carrier particles, the wall, the air flow and with each other was modelled using CFD–DEM and empirical correlations were proposed to describe the interaction of the small drug particles at the larger scales. At the meso-scale, the larger carrier particles and their interactions were modelled with the collisions modelled using a visco-elastic model to describe the local deformation at each point of particle–particle contact in conjunction with a model to account for cohesion. At the macro-scale, simulations of a complete prototype inhaler were carried out. By combining each of the scales, the total release efficiency of the fine particles could be obtained. LES instead of RANS was used to simulate the flow with a time-dependant inhalation flow rate. The results could also be used to study regions of recirculation, where carrier particles were trapped, and regions where fines adhere to the wall of the device. Nguyen et al. [38] employed the same approach in combination with an experimental investigation to study the performance of different device prototypes. A couple of mechanisms derived from experimental findings were incorporated to improve the predictive power of the model: (1) dispersion efficiency of the drug particles was reduced at a lower drug load; (2) a critical adhesion parameter was included to account for the irreversible sticking of fine particles to the carrier during the high shear mixing process; and (3) the application of surface energy distributions in analogy to adhesion force distributions. By integrating all three mechanisms, it was shown that the simulation model was able to predict the FPF of different formulations, devices and flow rates, with overall good agreement with experiments. Van Wachem et al. [39] recently carried out further simulations to account for the fragmentation of agglomerates due to agglomerate–agglomerate and agglomerate–wall impactions. The resolved micro-scale modelling results were used to construct a macro-scale discrete fragmentation model.

### 3.4. Modelling of Capsule-Related Effect

**Figure 5:**
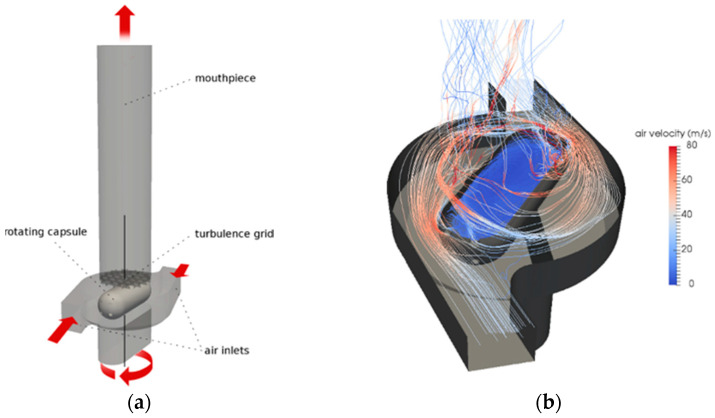
(**a**) Schematic of an Aerolizer^®^ inhaler with a pierced capsule and (**b**) streamlines in the capsule chamber. Reproduced/Adapted with permission from [10], Elsevier, 2019.

For capsule-based DPI, the presence, dimension and movement of the capsule could greatly influence the flow field in the inhaler, as well as the de-agglomeration and dispersion of particles. The capsule is initially placed in the capsule chamber of the DPI and pierced in a certain way before inhalation to allow particles to be fluidised and move out of the capsule (Figure 5a). During the inhalation, the high-speed air flow (normally around 60 L/min) from the inlet of the inhaler forces the capsule to rotate and collide frequently with the inhaler wall (Figure 5b). This would, for a certain degree, help the de-agglomeration process of the particle clusters and enhance the performance of the DPI. The drug emptying process normally takes about several seconds. It is a rather complex process, involving the interaction of solid capsule, particles and air flow. At an even higher air flow, for example, over 100 L/min, the shattering of the capsule may occur, which will further increase the complexity of the problem.

Existing studies in the literature mostly employed the combination of both experiments and simulations to gain an understanding of the effect of the capsule. Coates et al. [19] performed CFD analysis to determine the flow field in a commercial DPI device, Aerolizer^®^, with the presence of the capsule. In their modelling, a simplified capsule motion was assumed, where both the axis and the centre point of the rotation were fixed, and a constant angular velocity was employed. The angular velocity was not determined by simulations but was based on high-speed photography measurements. Numerically, the simulation was achieved by setting a rotating domain, where the capsule was housed, and a stationary frame, where the flow in the main body of the device was solved, and the two flow domains were linked via a transient rotor–stator model. The ejection of powder contained in the capsule through the capsule holes, as well as the particle de-agglomeration, were not modelled. The dispersion performance of the inhaler using different capsule sizes and powder loading methods were determined using a multi-stage liquid impinge. It was evidenced from the CFD resolved flow field that the presence of the capsule actually reduced the overall turbulence level. However, the dispersion analysis indicated that an initial de-agglomeration effect was provided by the capsule when the powder exited the small holes, which resulted in enhanced overall performance of the DPI, although this could not be proven directly by simulations.

Benque and Khinast [10] took a similar approach to capsule motion simulation and extended the CFD flow field to include the internal space of the capsule. The air velocities inside the capsule and at the capsule holes were examined, where the peak velocity could reach 35 m/s. Furthermore, the effect of collisions of the capsule with the inhaler walls on the powder discharge from the capsule was evaluated. DEM simulations were used to compare the powder retentions with and without capsule-inhaler collisions, in a given inhalation time. The results highlighted the importance of the collisions, which helped to break up particle clusters and allowed an easier powder discharge from the capsule.

Relevant studies are still scarce in the literature, due to the complexity of the problem that includes the motion, physical state and emptying behaviour of capsule, as stated by Huynh et al. [73]. Several issues have been identified, which may potentially affect their predicting capabilities of the available models. Firstly, the particle entrainment from a powder bed has not been well described. A complete model including all particles would result in an unrealistic computational cost due to the large number of particles involved. Apart from this, more detailed discussions on the effect of capsule geometries and the position of the holes are in need to improve the dispersion performance of the DPI device [74].

## 4. Application of Computation Modelling for Emerging DPI Designs

The therapeutic application of respiratory drugs has rapidly expanded beyond conventional indications, like asthma and chronic obstructive pulmonary diseases (COPDs), to include inhaled antimicrobials, vaccines and medications for systemic delivery. These emerging applications of inhaled therapy usually require high doses of the active agents (greater than 100 mg), like the inhaled antibiotic treatments, and are designed for patients with varied lung capacities. In this section, we will demonstrate the applicability of computational modelling in optimising the DPI designs for the delivery of high dose medication, to paediatric patients and personalised applications. Representative studies in this field are listed in Table 3.

### 4.1. Designing High-Dose DPIs

The properties and behaviour of low-dose and high-dose cohesive powder could be quite different so most high-dose formulations are engineered with little or no excipients using techniques like spray drying and supercritical fluid drying. Therefore, high-dose powders are usually highly cohesive, adhesive and compacted, with their dispersion largely relying on particle–particle and particle–wall collisions, which may cause considerable powder retention within the inhaler [80]. Therefore, innovative DPI designs are required for efficient delivery of such high dose powder to the lungs. 

The Twincer^™^ is a disposable device utilising the air classifier technology dispersing powders based on inertial forces for the delivery of 55 mg of milled colistimethate sodium without excipients [81,82]. Basically, the inhaler consists of three plate-like parts with various projections and depressions constituting the air flow passages. The formulation is stored in a pre-filled dose compartment for patients to administer in one inhalation manoeuvre. Small changes in the geometric details of the classifiers were reported to have a significant effect on the Twincer™ performance [83]. Therefore, de Boer et al. [27] employed CFD to predict flow and particle behaviour within Twincer™ to get a better understanding of design changes for optimisation. They found that the pressure drop across the inhaler had little impact on the flow split of the Twincer™ and the bypass channel does not contribute to the swirl within the classifier. Therefore, they proposed blocking the bypass channel would not affect the inhaler performance, instead, this would help to reduce the total flow rate at the same pressure drop, minimising the mouth and throat deposition. The particle trajectory results also indicated that particles of size ≥10 µm would retain in the classifier and help to sweep off adhering drug particles from the cylindrical walls of the classifier. Based on these computational observations, the bypass channel was removed in the development of the new member of the Twincer^™^ family, Cyclops™, and lactose sweeper crystals 150–200 µm were added to improve the dispersion of tobramycin [84].

TwinCaps^®^ is a single-use disposable inhaler designed by for the delivery of a total of 40 mg laninamivir octanoate marketed as Inavir^®^ for the treatment and prevention of influenza A and B infection in Japan. It comprises only two plastic parts: a body with the mouthpiece and a moveable shuttle containing two prefilled powder doses held in place by the body. CFD modelling of this device showed that the shuttle compartment design creates a strong bottom jet associated with high turbulent kinetic energy to disperse the powder [75]. The jet expansion through the restricted compartment design can also generate significant recirculation zones to increase the particle residence time within the inhaler to facilitate powder dispersion. Re-design of TwinCaps^®^ to achieve larger dose delivery (TwinMax^®^) was aided by CFD to include a few lateral air vents in the shuttle, forming pairs at various heights of the powder compartment, each pair providing a non-tangential admission of air. The flow patterns reveal that the new compartment design is capable of creating high flow velocity magnitudes at the bottom of the compartment and near the side walls to induce non-uniform axial and tangential flow components varying along the axial direction. The high-flow turbulent kinetic energy and high flow vorticity within the compartment effectively enhance powder dispersion for high dose drug delivery. The emitted dose and FPF for TwinMax were >90% and >40%, respectively, comparable to the TwinCaps^®^.

Overall, CFD has been well adopted by the industry as a useful tool in redesigning existing DPI devices to deliver a higher API dose.

### 4.2. Designing DPIs for Paediatric Patients

While the currently available DPIs have been very successful for adult patients, their use in children is challenging and there is a need to design children-specific or child-friendly inhalers. The upper airway depositional losses of 80% or higher is common in children [9,85]. Furthermore, there is significantly high inter-subject variability in paediatric patients. Bass et al. [85] identified the key problems associated with paediatric use of DPIs: oral administration via passives device operating on negative inhalation pressure, turbulence and jet momentum causing high depositional losses in the devices as well as in upper airway and relatively large and static aerosol size. In recent years, technology development is underway to address these challenges. Some of these include nose-to-lung aerosol administration (in place of conventional oral administration) using sufficiently small particles [86], active positive pressure devices [87] and using patient interfaces such as a mouthpiece or a nasal cannula to reduce turbulence and jet momentum effect without increasing particle deposition loss [88]. The development of such devices is being driven by a synergistic combination of CFD simulations and in vitro experiments. 

Bass et al. [76] developed a computational methodology and guidelines to model nose-to-lung aerosol administration to an infant and validated the model with in vitro experiments. The Reynolds number in the tube connecting to the nasal cavity was about 900. However, the flow path in the nasal passage was complex and could become turbulent. They compared the results obtained using a laminar and low Reynolds number *k-ω* turbulence model. They implemented a near-wall correction in the low Reynolds number *k-ω* turbulence model to incorporate anisotropic velocity fluctuations in the continuous phase and damp the wall-normal velocity to represent particle–wall interactions. While both the laminar and turbulent models predicted deposition fraction correctly, they suggested that the use of turbulence model is required to capture the flow field correctly. 

Positive pressure devices require an external gas source to aerosolise the powder. The gas may be delivered via an air syringe, ventilation bag or compressed air. Longest and co-workers developed a positive pressure air-jet DPI, operated with a ventilation bag or compressed gas supply. The air-jet DPI consists of a small diameter inlet passage, aerosolisation chamber and small diameter exit passage and gas at positive pressure passes from it and forms a turbulent jet within the aerosolisation chamber. Using CFD, they optimised the performance of the air-jet DPI [77,89]. 

Das et al. [90] performed CFD simulations in an idealised upper airway geometry for a five-year-old child to an adult with breathing conditions relevant to DPI inhalation. Based on the CFD simulations, they suggested the existence of a dimensionless curve for deposition in the conducting airways and found the deposition peak to occur at a Stokes number of 0.06 irrespective of age for DPIs. This guideline can be translated to an optimal aerosol size for a particular age group. 

In summary, the application of CFD combined with in vitro experiments is continuously increasing to design new DPIs or modify the existing devices for pediatric use.

### 4.3. Designing DPIs Independent of Patients’ Inhalation Manoeuvre

In conventional DPI devices, the drug dispersion is strongly dependent on the patients’ inhalation manoeuvre: when a patient generates a strong inhalation flow rate, faster drug entrainment from the powder bed would be expected, causing a completely different drug dispersion compared to a low-flow scenario. A recent trend of the DPI design is to develop next-generation devices that are independent of a patient’s ability and achieve consistent drug delivery performance [78]. The computational modelling approach is a powerful tool that has several significant advantages for the development of optimised device design, including its high efficiency and economic feasibility to deal with many design options in a wide range of flow rate, as well as its ability to provide a full picture of the flow field and relevant particle trajectories.

**Figure 6:**
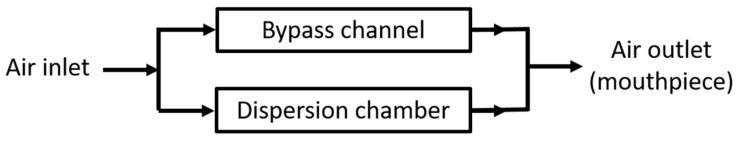
Schematic of a DPI device layout with a bypass channel to achieve flow independency.

Kopsch et al. [78,79,91,92,93] proposed a solution to achieve patient independent drug delivery by introducing a suitable amount of air bypass as a DPI design factor for controlling the powder entrainment rate. A fast computational method to determine the optimal amount of air bypass has been developed and implemented. The method was based on a two-phase Eulerian–Eulerian framework in which the drug particles were modelled as a second continuous phase and a volume fraction (*α*) was introduced to keep track of the local fraction of drug phase. An example generic design of this type of devices was given in [78], and the simplified drawing and schematic device layout are shown in Figure 6. The total incoming air flow generated by the patient split into two streams, one flowed through the entrainment compartment and the other through the bypass element. Different device designs would result in different flow resistances and hence different bypass ratios. The drug release performance was strongly correlated with the bypass ratio, which minimised its variability between different inhalation manoeuvres.

Kopsch et al. [91] also developed a cost function and used an optimisation algorithm to find the minimum of the cost function, which was equivalent to the most preferred design to achieve independence from the patient’s respiratory function. Based on a simple DPI device, five design variables were proposed, which represented five different dimensional parameters of the device. During the optimisation process, the Eulerian–Eulerian solver was firstly used to simulate the drug release through the device at different flow rate profiles. A cost function was then calculated, and a new set of design parameters were determined using the optimisation algorithm. The iteration continued until a minimum cost function was obtained.

Kopsch et al. [79] demonstrated experimentally that the proposed fast computational method could accurately predict the entrainment of the dry powder formulation in DPIs. In their experiments, the chosen entrainment module was filled with lactose powder and the entrainment process was recorded with a high-speed camera. The mass of drug released as a function of time was determined from the videos and compared with the modelling results. It showed that the predictions agreed strongly with experimental results for powders with a relatively low fraction of fines, in which the cohesive forces between particles were not relevant.

As indicated above, studies in this field are still at an early stage. More effort is required for the development of more reliable modelling approaches and innovative DPI design concepts to achieve enhanced drug delivery performance with good independence from the patient’s inhalation ability.

## 5. Challenges and Future Works

This review indicates that the numerical modelling of flows and particle behaviours in the DPI has become a useful tool to predict powder dispersion for both carrier-based and carrier-free drugs. RANS is currently the most popular CFD method to resolve a turbulent flow field in the DPI, while the LES has been increasingly used due to the rapid development of modern computing technologies. Compared to the modelling of the flow field, it is far more challenging to carry out high-level prediction of particle behaviours and the coupling effect between the flow and particle in DPI applications. To avoid simultaneously considering a vast number of particles and their interactions, several recent studies have been proposed to adopt a multi-scale modelling approach, so that the micro-scale particle de-agglomeration and device-level flow-particle interaction can be solved separately. Nevertheless, significant improvement is still required to further enhance the capability of the model to capture all important particle behaviours and, on the other hand, simplify the particle-related force model to reduce the computational demand. Since the most advanced DPI can only achieve an aerosolisation efficiency as low as 15–40% [5], there is still a great potential to further optimise the device design using computational approaches. 

The following research directions are proposed, and it is expected relevant efforts would extend the application of modelling techniques in the DPI design:

- More direct comparisons between simulation and experimental results are required to enhance the reliability of the computational approaches. Most existing experimental validations only involve the measurement of overall particle dispersion using standardised impactors [16,26,36], where the information of particle spatial distribution is absent. A laser imaging device can be used to visualise the shape of the powder cloud [65,94] but is unable to distinguish the different particle sizes in the cloud. Experimental techniques that can carry out measurement of both particle size and spatial distribution simultaneously are required for more reliable validation of the most recent modelling approaches.
- Most existing modelling studies for DPI application have assumed both carrier and fine particles to be a sphere with a smooth surface. In reality, the exact shape of the particle depends on how the powder is prepared: the spray-dried particle is close to spherical and regular in shape but with extremely rough surface whist the milled and sieved particle was very much non-spherical and irregular with a relatively smoother flat surface [15]. Both the shape and the surface roughness of the particle would strongly influence the particle–particle forces and dispersion behaviours. Tamadondar and Rasmuson [66] indicated that the particle dispersion efficiency correlated with the surface asperity. Ali et al. [95] briefly discussed the influence of particle shape to its flowability; Ohsaki et al. [96] studied the deposition behaviour of rod-like particles with large aspect ratios. More efforts are required to include relevant effects in existing modelling approaches.
- Multi-scale approaches have been adopted to simulate the carrier-based particle behaviours in DPIs. The re-attachment of fines, which were previously detached, to moving carrier particles have been considered [12]. However, the possible agglomeration, as well as wall deposition of fines, after they are detached from the carrier and dispersed in the air flow, has not been well examined. It is expected that a large residence period of dispersed fines in the DPI would cause agglomeration and the deposition of fines therefore reduce the FPF at the outlet of the device. Future studies need to take this into account by including additional mechanisms to the existing multi-scale models.
- For capsule-based DPIs, most modelling studies have not included the discharge process from the capsule since it involves the entrainment of a large number of particles from the powder bed inside the capsule, which could result in unrealistically large computational cost. However, the flow inside the capsule is highly turbulent and may cause a considerable level of fine particle dispersion in an early stage of the inhalation. This could potentially affect the overall dispersion performance of the DPI. Several recent studies [77,89,97] have suggested that the particle dispersion in the capsule was strongly correlated with the parameters which were used to describe the turbulence of the flow. This can be further explored for the development of a low-computational-cost modelling approach considering the effect of particle discharging from the capsule.

## 6. Conclusions

This study has introduced and critically reviewed various modelling methodologies used for predicting fluid and particle behaviours in DPIs. Most recent modelling studies have been summarised, which can be classified into three categories: (1) particle trajectory modelling, (2) particle–particle interaction modelling and (3) the latest multi-scale modelling. The application of modelling tools to the development of emerging DPIs has been discussed, such as those with the features of high-dose, pediatric patient compatibility and independency of patients’ inhalation efforts. Opinions on the challenges and future research directions have been given based on the author’s opinion.

RANS is currently still the most popular CFD method for the flow field simulation in DPIs while the feasibility of LES is improving rapidly. The multi-scale modelling approach has been increasingly used to predict device-scale particle dispersion. Compared to conventional devices, the use of computational modelling for the design of various emerging DPI devices is still very limited, although some great advantages have been realised based on available results in the literature. To further enhance the performance and capability of modelling tools, high-quality experimental measurement of the particle dispersion, which includes the information of both particle size and spatial distribution, is highly regarded for more reliable validation of the cutting-edge modelling methods.

## Figures and Tables

**Table 1:** Comparison of different computational fluid dynamics (CFD) methods for DPI simulations.

CFD Method	Turbulent Model	Modelling Accuracy	Computational Cost	DPI Applications
Reynolds-averaged Navier–Stokes (RANS)	Standard *k-ε*	Medium	Low	Aerolizer [14], NGC (new generation Cyclohaler) [14], ACTIVAIR [15]
	Standard *k-ω*	Medium	Low	-
	*k-ω* SST	Medium	Low	Aerolizer [8,11,16,17,18,19,20], Turbuhaler [13,21,22], Handihaler [20], standardized entrainment tube [23,24,25]
	RNG *k-ε*	Medium	Low	Modified Cyclohalers [26]
	Realisable *k-ω*	Medium	Low	Twincer [27], Cyclohaler [28], Handihaler [28], Accuhaler [29], Multihaler [29]
	Reynolds stress model	Relatively high	Medium	Aerolizer [30,31,32,33,34,35,36], T-pipe [37]
Large eddy simulation (LES)	-	High	High	L-shaped channel [12,38,39]
Direct numerical simulation (DNS)	-	Fully resolved	Extremely high	-

**Table 2:** Summary of numerical studies of flow field and particle tracking for DPI applications.

Reference	Inhaler Type	Flow Simulation Method	Particle Tracking Method	Experimental Validation	Modelling Platform	Objectives
Coates et al. [8,16,17,18,19]	Aerolizer^®^	RANS with *k-ω* SST model	Particle trajectory modelling	Laser Doppler Velocimetry, multistage liquid impinger	ANSYS CFX	Study the effects of grid structure, mouthpiece length, capsule type, air flow, air inlet size and mouthpiece geometry on DPI performance
Milenkovic et al. [13,21,22]	Turbuhaler^®^	RANS with *k-ω* SST model, LES	Particle trajectory modelling	N/A	ANSYS Fluent	Verification of numerical method; study the effect of dynamic flow and geometry modification to DPI performance
Wong et al. [23,24,25]	Standardized entrainment tube	RANS with *k-ω* SST model	Particle trajectory modelling	Laser diffraction (Spraytec particle sizer)	ANSYS CFX	Study the effect of impaction, turbulence and grid structure on the aerosolisation and break-up of pharmaceutical agglomerates
Moskal and Sosnowski [14]	Aerolizer^®^, NGC (new generation cyclohaler^®^)	RANS with standard *k-ε* model	Particle trajectory modelling	Direct flow visualization using high-speed camera	ANSYS Fluent	Study the effect of mouthpiece length for fine particle delivery
Calvert et al. [50,51,52]	N/A	No turbulent model used	Particle–particle interaction	Laser differaction (Mastersizer 2000)	PFC3D	Study the effect of surface energy, aggregate diameter and flow velocity to the dispersion of loose aggregate
Suwandecha et al. [26]	Modified Cyclohalers^®^	RANS with RNG *k-ε* model	N/A	8-stage Andersen Cascade Impactor	ANSYS Fluent	Develop new inhaler devices via computer-aided design
Longest et al. [53]	Standard and modified flow passages	RANS with LRN *k-ω* model	Particle trajectory modelling	Next Generation Impactor	ANSYS Fluent	Determine the aerodynamic factors which were most responsible for de-aggregating carrier-free powders
Sommerfeld and Schmalfuß [11]	Aerolizer^®^	RANS with *k-ω* SST model	Particle trajectory modelling	N/A	OpenFOAM	Develop a detachment model related to fluid stresses and wall collision
de Boer et al. [27]	Twincer^®^	RANS with Realisable *k-ω* model	Particle trajectory modelling	Next generation impactor and multi-stage liquid impinger; laser diffraction	ANSYS Fluent	Evaluate the high-dose disposable DPI and study the effect of design modification on particle behaviour
Donovan et al. [20]	Aerolizer^®^, Handihaler^®^	RANS with *k-ω* SST model	Particle trajectory modelling	Cascade impactor	ANSYS Fluent	Study the effect of both carrier particle physical properties and inhalation device on the performance of DPI
Shur et al. [28,29]	Cyclohaler^®^, Handihaler^®^, Accuhaler^®^, Multihaler^®^	RANS with Realisable *k-ω* model	Particle trajectory modelling	High-speed laser imaging; next generation impactor	ANSYS Fluent	Identify the key device attributes and evaluate the effect of their modifications on the aerosolization performance
Thornton et al. [54]; Kafui and Thornton [55]; Mishar and Thornton [56]; Thornton and Liu [57]	N/A	N/A	Particle–particle interaction	N/A	TRUBAL, GRANULE	Study the impact of agglomerate with a target wall and the effect of impact velocity and bond strength to fracture patterns
Tong et al. [31,32,34,35,37]	Aerolizer^®^, impact throats, T-pipe	RANS with Reynolds stress model	Particle–particle interaction	N/A	ANSYS Fluent	Study the effect of velocity, angle, particle size, on breakage of agglomerate and dispersion performance of DPI
Tong et al. [30]; Leung et al. [36]	Aerolizer^®^	RANS with Reynolds stress model	Multi-scale modelling	Next generation impactor	ANSYS Fluent	Study carrier-based dry powder aerosolisation in DPIs
van Wachem et al. [12,39]; Nguyen et al. [38]	L-shaped channel	LES	Multi-scale modelling	Next generation impactor	N/A	Study carrier-based and carrier-free agglomerate fragmentation and DPI dispersion performance
Lee et al. [15]	ACTIVAIR^®^	RANS with standard *k-ε* model	Particle trajectory modelling	Particle image velocimetry; Anderson cascade impactor	ANSYS Fluent	Compare the particle dispersion pattern of a spiral and non-spiral mouthpiece designs
Yang et al. [58,59,60]	N/A	No turbulent model used	Particle–particle interaction	N/A	N/A	Study air flow and particle-wall impact-induced dispersion of carrier-based dry powder

N/A: not available.

**Table 3:** Representative computational modelling studies for emerging DPI designs.

Inhalers	Powder System	Insights from Modelling Results	Development Status	Reference
Development of new inhalers for high dose medication				
Twincer™	Milled Colistimethate sodium (55 mg)	Pressure effect is negligible on flow split Bypass channel does not contribute to swirl	Lead to the design of Cyclops™ for the delivery of 50 mg tobramycin	de Boer et al. [27]
Twincer™	Laninamivir octanoate (40 mg)	High turbulent kinetic energy and recirculation zones associated with shuttle compartment design	Lead to the design of TwinMax^®^ to delivery 80 mg powder	Fernandes et al. [75]
Development of new inhalers suitable for paediatric patients				
Nose-to-lung (N2L) aerosol device	Not specified	CFD and in vitro experiment showed low inertial and turbulence depositional loss when aerosol with an aerodynamic diameter of ∼1.5 μm through an infant nasal cannula interface	N2L is suitable for lung delivery to infants when correctly sized aerosols are used	Bass et al. [76]
Active positive pressure air-jet DPI	Spray-dried albuterol sulfate, mannitol, leucine and Poloxamer 188	Strong secondary flow was evident between the inlet jet and capsule Optimal length of the outlet capillary was correlated to the turbulent kinetic energy	An optimised paediatric air-jet DPI design suitable for 5–6-year-old children was identified	Bass et al. [77]
Development of inhalers independent of patients’ inhalation manoeuvre				
Generic entrainment modules	Sieved and milled *α*-lactose monohydrate	Modelling approach combining a cost function can identify the optimal amount of air bypass accurately	Conceptual design with air bypass was verified	Kopsch et al. [78,79]
